# A Study of the Three-Body Abrasive Wear Resistance of 5V/5Nb-5Cr-5Mo-5W-5Co-Fe Multicomponent Cast Alloys with Different Carbon Percentages

**DOI:** 10.3390/ma16083102

**Published:** 2023-04-14

**Authors:** Riki Hendra Purba, Kazumichi Shimizu, Kenta Kusumoto, Yila Gaqi, Mohammad Jobayer Huq

**Affiliations:** 1 Department of Production Systems Engineering, Muroran Institute of Technology, 27-1 Mizumoto, Muroran City 050-8585, Japan; shimizu@mmm.muroran-it.ac.jp (K.S.); kusumoto@mmm.muroran-it.ac.jp (K.K.); 20096003@mmm.muroran-it.ac.jp (Y.G.); 21096513@mmm.muroran-it.ac.jp (M.J.H.); 2 Department of Mechanical Engineering, University of Sumatera Utara, Medan 20155, Indonesia

**Keywords:** abrasive wear, multicomponent white cast iron, carbon percentage

## Abstract

Since it is well known in the literature that transition metals can form extremely hard carbides and effectively strengthen a material’s matrix, recently, some of them, such as V, Nb, Cr, Mo, and W, have been simultaneously added to cast iron. In addition, it is common to add Co to cast iron to strengthen the material’s matrix. However, the wear resistance of cast iron can also be considerably affected by the addition of C, which is rarely discussed in the literature by the experts. Therefore, the effect of C content (1.0; 1.5; 2.0 wt.%) on the abrasive wear behavior of 5 wt.% V/Nb, Cr, Mo, W, and Co alloys was investigated in this study. An evaluation was conducted using a rubber wheel abrasion testing machine in accordance with ASTM G65 with silica sand (1100 HV; 300 μm) as abrasive particles. The results show that plural carbides (MC, M_2_C, and M_7_C3) precipitated on the microstructure of the material, which is not unlike the behavior of other types of carbides as the quantity of C increases. The hardness and wear resistance properties of 5V-5Cr-5Mo-5W-5Co-Fe and 5Nb-5Cr-5Mo-5W-5Co-Fe multicomponent cast alloys increased as the quantity of C increased. However, we observed no significant difference in the hardness between the two materials with the same C additions, while 5Nb presented better wear resistance properties compared to the 5V sample due to the larger size of NbC compared to VC. Therefore, it can be determined that, in this study, the size of the carbide plays a more important role than its volume fraction and hardness.

## 1. Introduction

In the literature, it has been estimated that approximately 23% of the world’s total energy was invested in solving friction and worn-components problems as a result of wear phenomena in the year 2017 [1]. Therefore, the development of materials with superior wear resistance properties is being conducted by numerous researchers at present. Several forms of wear activity exist, such as abrasive, fritting, erosive, adhesive, and corrosive. However, abrasive wear, especially the three-body type, can cause more problems to materials compared to the others [2,3]. Three-body abrasive wear can be explained as the surface damage caused by the presence of hard particles between two surfaces during the process of relative motion [4]. It can occur in several parts of engineering machinery. For example, abrasion often occurs on transportation equipment when iron ore is shipped from stock areas to blast furnace feeders in the steel-making process. Once it occurs on the surface of this important equipment, it significantly increases the cost of steel production [2,4]. Therefore, it is very important to maintain the service life of this particular part of the machine. In addition, it can also occur in equipment, such as metal crushers, roll mills, excavators, etc. Therefore, this study attempts to overcome this issue by discovering superior three-body abrasive wear resistance materials.

Traditional high-chromium white cast iron (HCCI) consists of 12–30 wt.% Cr and 2.0–4.3 wt.% C, and it has been utilized under severe wear conditions since the early 1980s [5,6,7]. Zum-Gahr et al. [8] stated that the outstanding wear resistance of HCCI occurs due to the crystallization of the hard phase (M_7_C_3_) in the microstructure. In general, a higher level of wear resistance can be achieved by an increase in the number of M_7_C_3_ carbides. In addition, it is well known in the literature that the properties of hardness and abrasion also considerably affect the wear resistance of these alloys [5,9,10]. Nevertheless, it is important to note that the presence of M_7_C_3_ carbides in the microstructure makes it susceptible to cracking due to its low level of strength resulting in its very limited application [11,12,13]. Several attempts have been made by experts in the area to overcome this problem, for example, by modifying the matrix, the orientation of the M_7_C_3_ carbides, and refining the size of the carbides. It is also well known that HCCI with a martensite matrix has a higher level of abrasion resistance than austenite or ferrite and pearlite [14,15,16]. In addition, the precipitation of secondary carbides during the destabilization heat treatment process can also strengthen the matrix of HCCI providing a positive contribution to the abrasive wear resistance property [15]. In a study, Coronado [17] modified the carbide orientation of M_7_C_3_ by maintaining the time that was required for its solidification process and determined that the strength of this alloy, as well as its wear resistance, increased. The addition of Ti, Nb, and V elements to refine the size of the M_7_C_3_ carbide has also been proposed by other researchers [18,19,20]. In the literature, it is commonly understood that these transition metals first react with C during the melting process to form TiC, NbC, or VC. Then, when the melt temperature reaches the M_7_C_3_ precipitation temperature, smaller M_7_C_3_ carbides precipitate inside the microstructure resulting in greater carbide strength and an improved level of wear resistance. Meanwhile, the effects of other transition metals, such as Mo and W, have also been reported in the literature. The influence of the addition of Mo to HCCI was also investigated by Shimizu et al. [21]. It was concluded that the wear resistance of the alloy improved by approximately 28–36% due to the presence of Mo_2_C carbides in the microstructure. Furthermore, Anijdan et al. [22] studied the effect of W on HCCI and revealed that the wear resistance property increased due to the increase in the matrix’s strength and the formation of W_2_C carbides.

However, the majority of the abovementioned efforts involve only one or two types of transition metals. Recently, some researchers have added other transition metals to white cast iron, calling it multi-component white cast iron (MWCI). This new type of alloy performs better than HCCI during both abrasive and erosive wear conditions. It is associated with the precipitation of extremely hard carbides, MC, M_7_C_3_, and M_2_C, in the microstructure of an alloy, despite having a lower CVF than HCCI [23,24,25]. The wear resistance of MWCI can also be enhanced by the addition of the Co element. It is well known in the research that Co adds strength to the solid solution of MWCI containing 5 wt.% Cr, V, Mo, and W with 2 wt.% C [26]. However, the wear resistance of this alloy rapidly decreases once the quantity of Co exceeds 5 wt.% due to the strength-reduction effect. Unfortunately, the majority of the published articles only study the effects of transition metals. Meanwhile, the microstructure of the material is also considerably affected by the addition of C. Indeed, it has been reported in the literature that hypoeutectic HCCI (less than 3 wt.% C) presents better wear resistance properties compared to hypereutectic alloys [27,28]. However, an investigation conducted on the effect of C content on the wear performance of hypoeutectic MWCIs has not yet been reported in the literature.

Therefore, in this study, we evaluate the wear resistance properties of 5V/5Nb-5Cr-5Mo 5W-5Co-Fe multicomponent cast alloys with different carbon percentages under three-body abrasive wear conditions to expand our knowledge of the materials and obtain materials with superior wear resistance qualities. Our observations are related to the microstructure and hardness of the materials using optical microscopy, SEM-EDS, XRD, ImageJ, and the Vickers hardness test.

## 2. Materials and Methods

### 2.1. Preparation of Alloys

**Figure 1:**
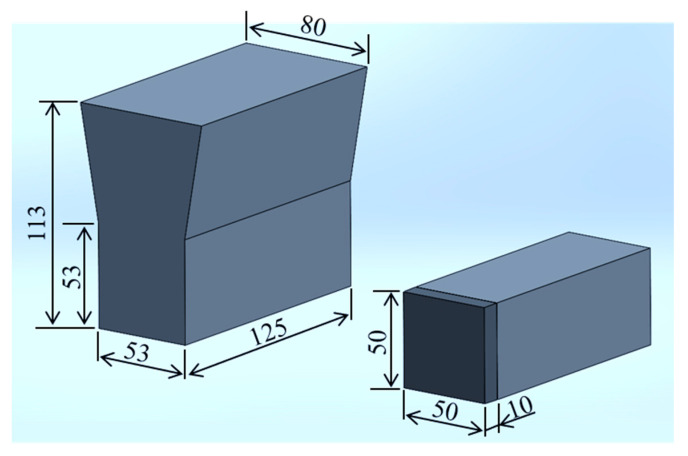
Y-shaped ingot of cast alloy with specimen dimensions.

The alloys were designed using two kinds of MWCIs containing 5 wt.% Cr, Mo, W, and V and 5 wt.% Cr, Mo, W, and Nb, with three different C percentages (1, 1.5, and 2.0 wt.%). In total, we produced six types of material (hereafter referred to as 1C-5V, 1.5C-5V, 2C-5V, 1C-5Nb, 1.5C-5Nb, and 2C-5Nb). The chemical composition of the alloys was measured using SPECTROLAB (AMATEK, Inc., Berwyn, PA, USA) and the result is presented in Table 1. The manufacturing process of the materials was introduced in a previous study we conducted [29]. First, 50 kg of raw material was melted using a high-induction furnace and then poured into a Y sand block mold (Figure 1). Then, a high-speed precision-cutting machine (Refinetech Co., Ltd., RCA-234, Kanagawa, Japan) was used to cut the specimens into dimensions of 50 mm × 10 mm × 10 mm. The cutter allowed for a coolant to automatically flow through it to prevent the alteration of the alloy’s microstructure owing to the friction phenomena that can occur during the process.

As previously explained, the austenite matrix transforms into martensite during the heat treatment process, which effectively increases the strength and wear resistance of the alloy. Moreover, the secondary carbide is formed during this hardening process, which reinforces the martensite matrix. In general, the destabilization heat treatment of MWCI occurs as the alloy is heated at 1173–1423 K for several hours and is then quenched using the air-force-cooling method. The process is continued during the tempering process, where the alloy is re-heated at 693–813 K and then cooled using the air-force-cooling method [30,31,32]. Therefore, all the alloys were pre-heated at 1323 K for one hour and then quenched using the air-force-cooling method. Subsequently, the product was tempered after being heated at 798 K for three hours and then cooled to room temperature using the air-force-cooling method. The surface of the alloys was polished using a grinder machine (GS52PF; Kuroda Seiko Co., Ltd., Kanagawa, Japan) to ensure that the surface roughness (Ra) of each alloy was uniform (approximately 0.21 µm). In addition, the ingot produced was cut to the dimensions of 10 × 10 × 10 mm in order to perform our investigation of the product’s microstructure. Then, a small, cubic plate was polished with silica sandpaper from 120, 400, 600, to 1200 P. Subsequently, the polishing was followed using 9, 3, and finally 1 μm of diamond suspension paste.

### 2.2. Microstructure Evaluation

To evaluate the microstructures of the specimens, they were first etched for approximately 5 min in a 3% nitric acid alcohol solution (Nital) at room temperature. OM; Eclipse LV150N, Nikon, Tokyo, Japan was used to observe the microstructures of each alloy. Scanning electron microscopy (SEM + EDS; JSM-6510A, JEOL, Tokyo, Japan) was used to determine the types of carbide, CVF and also the distribution of added elements. SEM was performed with a specimen stage: eucentric large-specimen stage, X: 80 mm, Y: 40 mm, Z: 5 mm to 48 mm; electrical image: shift ± 50 μm; and wavelength dispersive (WD: 9–20 mm) on an accelerating voltage of 20 kV. A factory pre-centered filament was used in the SEM. In addition, the carbide area on the SEM image was first colored with paint to enhance the contrast between the carbide area and the matrix. Then, the images were imported into ImageJ to perform the image binarizing process to calculate the CVF and carbide size. Additionally, spot or point analyses were performed in over 10 random locations in the SEM microphotograph to determine the stoichiometry or carbide type. Furthermore, the microstructure phase was analyzed using X-ray diffraction (XRD, Ultima VI Pro, Rigaku, Japan). This analysis was conducted with 2θ at 30–90 deg. The XRD machine used a Cu tube with a current value of 20 mA and 40 kV voltage. The X-ray beam dimension was 0.3 × 5 mm. The PDXL2 database was used to determine the XRD peak.

### 2.3. Measurement of Materials’ Hardness Level

The microhardness (matrix) property was measured using Vickers hardness tests (Future-Tech Co., Ltd.: FM-300, Kanagawa, Japan) with a 4.9 N load. Additionally, the microhardness (matrix and precipitated carbide) was measured using Vickers hardness tests (Future-Tech Co., Ltd.: FV-800, Kanagawa, Japan) with a 294 N load. The dwelling time for each measurement was approximately 15 s. The average value of 12 repetitions for each measurement was used to perform the analysis.

### 2.4. Abrasion Wear Test

**Figure 2:**
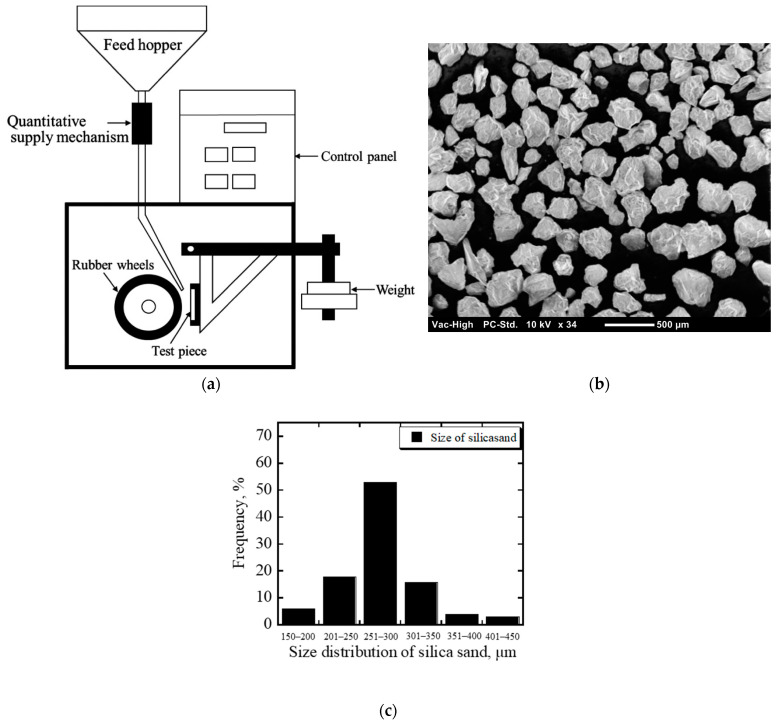
(**a**) Schematic drawing of the abrasion machine test; (**b**) image of the abrasive particle; (**c**) abrasive particle distribution size.

The abrasion wear performance of MWCI was investigated using a rubber wheel three-body abrasive wear machine test (ASTM G65 standard). The test condition was conducted with 196 N loads and a 1.2 m/s sliding speed for 6 min at room temperature. Silica sands (97.99% SiO_2_) with approximately 300 µm of the average value and a 1100 HV hardness value were used. Abrasive particles were poured from the hopper into the gap between the specimen’s surface and the wheel at a speed of approximately 4.2 g/s. The results of the three-body abrasive wear machine test and silica sand are presented in Figure 2. The average values of the data obtained from the repetition test, repeated six times, were used to perform the abrasive wear analysis. Equation (1) was used to calculate the wear rate of each MWCI:

Wear rate = ∆m/πdtn(1)

where ∆m is the mass of material loss (kg), d is the diameter of the wheel (m), t is the time (s), and n is the rotation speed (rpm).

Moreover, the Optelics hybrid (MC2000 Lasertech Co., Ltd., Yokohama, Japan) was used to obtain the depth of the scars of the tested specimens. The wear mechanism of each MWCI was observed via the worn surface and cross-section view. The most abraded worn surface and cross-section areas, which were cut into the dimensions of 10 × 10 × 10 mm, were embedded in epoxy resin. This small, cubic plate used for the cross-section analysis was first polished using silica sandpaper sizes ranging from 120, 400, 600, and 1200 P. Subsequently, the polishing process was followed by the use of 9, 3, and finally 1 μm diamond suspension pastes.

## 3. Results and Discussion

### 3.1. Microstructure Characteristics and Hardness

**Figure 3:**
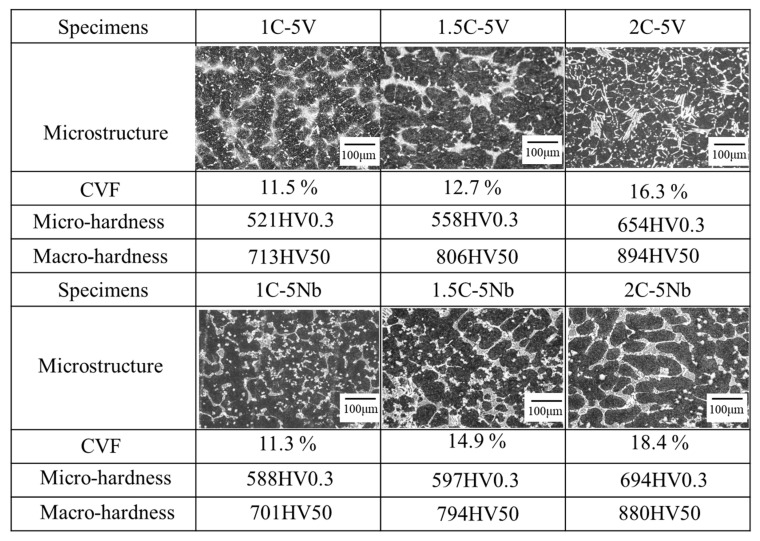
The microstructure, CVF, and Vickers hardness test of the alloys.

It is well known in the literature that the microstructure of alloys has important contributions to their wear evaluation results. Therefore, the effect of C on the microstructures of 5V-5Cr-5Mo-5W-5Co-Fe and 5Nb-5Cr-5Mo-5W-5Co-Fe multicomponent cast alloys was investigated in this study. The microstructures of the six alloys are presented in Figure 3. It shows that the microstructures of the alloys consist of carbides (lighter areas) and matrices (dark areas). In the case of the 5V alloy, the carbide volume fraction (CVF) increased as the percentage of C increased. The same trend is also evident in the case of 5Nb. Due to the high affinity of C atoms with each added transition metal, the higher percentage of C added to the molten iron would naturally form higher CVFs in the microstructure during the solidification process. This phenomenon might be the reason for the increase in CVFs as the quantity of elemental C is increased in both alloys. However, no significant difference was observed between 5V and 5Nb with the same addition of C. This was due to the addition of the same quantity of transition metals.

The strength of each alloy is presented in the figure presented below. It shows us that the microhardness (matrix) and macrohardness (matrix and carbide) properties of the 5V alloys increase as the percentage of element C increases. This condition also occurs in the case of 5Nb alloys. In addition, the strength properties of the 5V alloys were relatively the same as those of the 5Nb alloys with the same quantity of C. This means that the CVF corresponds to the strength of each alloy.

**Figure 4:**
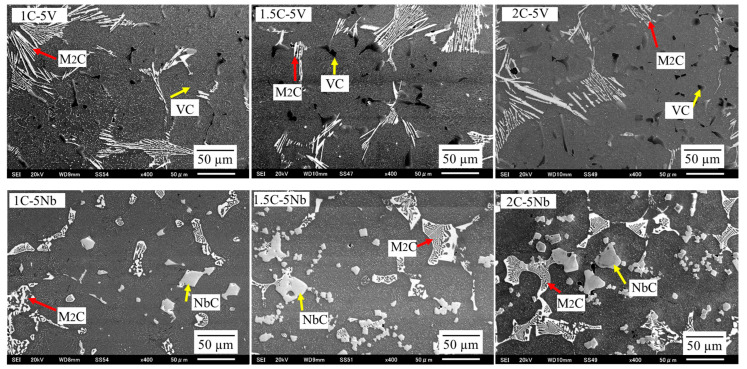
The microstructure of each alloy obtained by SEM. The VC (mainly globular shape) and M_2_C (fiber-like shape) carbides are precipitated on the microstructure of the 5V alloy. Meanwhile, the NbC (rectangular shape) and M_2_C (fishbone-like shape) carbides are deposited on the microstructure of the 5Nb alloy.

The type of carbides can be determined through a point analysis in SEM with more than 10 repetitions of the test being performed, as shown in Figure 4. In the case of the 5V alloy, a deposition of VC and M_2_C carbides where the letter M represents Mo and W is evident. The amount of VC seems to be greater as the percentage of C content increases. This condition resulted in an increase in the CVF. However, the VC size is relatively the same, which is approximately 5~10 μm. Meanwhile, there is no considerable difference evident in the M_2_C carbides. The shape of VC appears globular or plate-like, while M_2_C appears fiber-like. However, there is no visible difference in the shape of the carbides as the level of C increases. In addition, it was difficult to detect the Cr carbide using point analysis, which might have been due to the reduced additions of Cr.

In the case of 5Nb alloys, the precipitation of NbC and M_2_C occurred, where the location of the letter M was mostly occupied by Mo and W. Similar to VC, the value of NbC seems to be higher as the quantity of C content increases leading to a higher CVF, as previously described. The shape of NbC is rectangular and no considerable difference in the shape of this carbide occurs as the C content increases. The size of the NbC carbide is also similar, measuring approximately 20~30 μm, even though the amount of C increases. This means that the size of NbC is larger than VC owing to the larger atomic radius. In addition, we observed no considerable difference in the amount of M_2_C as the percentage of C increased. However, the shape of the M_2_C carbide appeared fishbone-like, which is unlike the 5V alloys, despite it possessing the same type of carbide. Thus, it can be determined that the shape and size of the precipitated carbides are strongly influenced by the overall chemical compound of the material.

**Figure 5:**
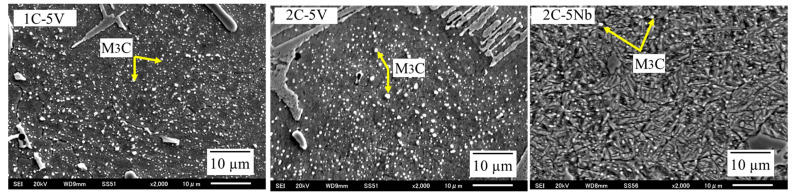
Higher-magnification microphotograph of the representative alloys. The precipitation of the secondary carbide (M_3_C) and martensite matrix (needle-like) on the microstructures of 1C-5V, 2C-5V, and 2C-5Nb.

In general, the austenite matrix would mostly transform into martensite during the destabilization heat treatment process (quenching and tempering). However, a small amount of austenite would remain in the microstructure of the white cast iron. In addition, a secondary carbide would also precipitate where its type and shape are strongly influenced by the heat treatment process (cooling rate or cooling method) and the chemical composition of the white cast iron [31,33,34]. In this study, the matrix of each alloy appeared to be needle-like, as shown in Figure 5. This means that martensite is the main matrix in which no significant difference was observed between the two alloys (5V and 5Nb). The retained austenite was quite difficult to obtain, which was a result that was different from the previous studies [33,34]. The difference in the results may have been due to the different destabilizing heat treatment processes used on the materials. The presence of secondary carbides in the matrix can also be observed in the figures. The M_3_C carbide was obtained by the point analysis technique on SEM-EDS. The M_3_C carbide is spherical in shape and very small in size (approximately 0.5–1 μm). In addition, the shape and size of M_3_C are very similar to those of 5V and 5Nb alloys.

**Figure 6:**
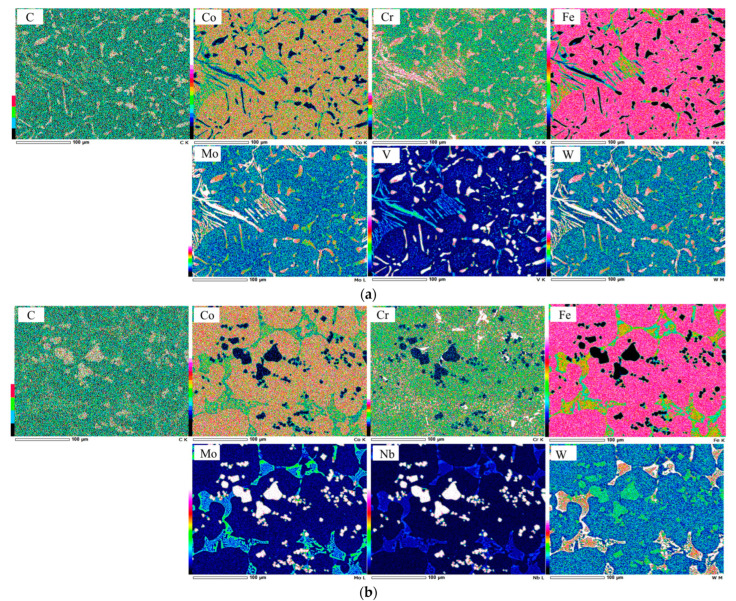
Elemental distribution of each transition metal on the microstructure. (**a**) 2C-5V alloy and (**b**) 2C-5Nb alloy.

Figure 6 presents the distribution of each added element in the microstructure. It can be observed that V is mostly embedded in VC carbides, and Mo or W occupies M_2_C carbides in 5V alloys. However, Mo was simultaneously embedded with elemental Nb to form NbC and W to form M_2_C carbides. Since Mo also occupied the area of the NbC carbide, this might have been the reason for the larger size of the carbide compared to VC. Meanwhile, Co and Fe are mostly embedded in the matrix area.

**Figure 7:**
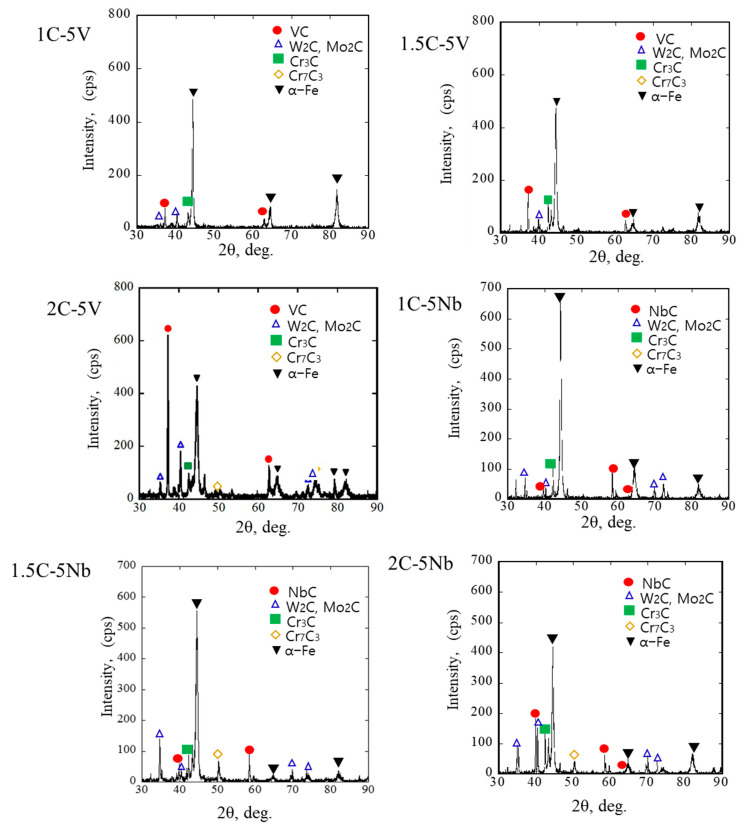
X-ray diffraction (XRD) patterns for each studied alloy.

To obtain more detailed microstructure information, the investigation was continued using XRD. The results of each alloy are depicted in Figure 7. In the case of the 5V alloy, there were several types of indexed carbides. These included VC, W_2_C or Mo_2_C, Cr_7_C_3_, and Cr_3_C carbides. It can be observed that the peaks of VC, W_2_C or Mo_2_C, and Cr_7_C_3_ increase as the amount of C increases in the case of the 5V alloy. This might be due to the better opportunity of added transition metals with more C atoms to form VC, W_2_C or Mo_2_C, and Cr_7_C_3_ carbides. However, no peak shifting was identified as the amount of C content increased. In addition, only an alpha (α) phase was evident in the results, indicating that martensite is the main material matrix type. No differences in the carbide or matrix were observed as the percentage of C increased. In the case of 5Nb, NbC, W_2_C or Mo_2_C, Cr_7_C_3_ and Cr_3_C, and Cr_7_C_3_ carbides were present in the microstructure. Similar to the 5V alloy, the XRD peak of 5Nb alloys presented the same behavior where the peaks of NbC, W_2_C or Mo_2_C, and Cr_7_C_3_ increased as the C content increased. The higher peak of the carbides as the C content increases might contribute to the increment in the CVF, as previously explained. Moreover, only the alpha (α) phase was detected on the microstructure of 5Nb alloys. Meanwhile, the presence of retained austenite was difficult to detect in 5V and 5Nb alloys, which might be due to its very little amount. This means that the XRD data are in good agreement with the SEM-EDS data, as previously described.

To determine the influence of all the microstructures and the strength of each alloy on the wear properties, they were evaluated under three-body abrasive wear conditions at room temperature, which are described in the subsequent section.

### 3.2. Three-Body Abrasive Wear Characteristics of the Alloys

**Figure 8:**
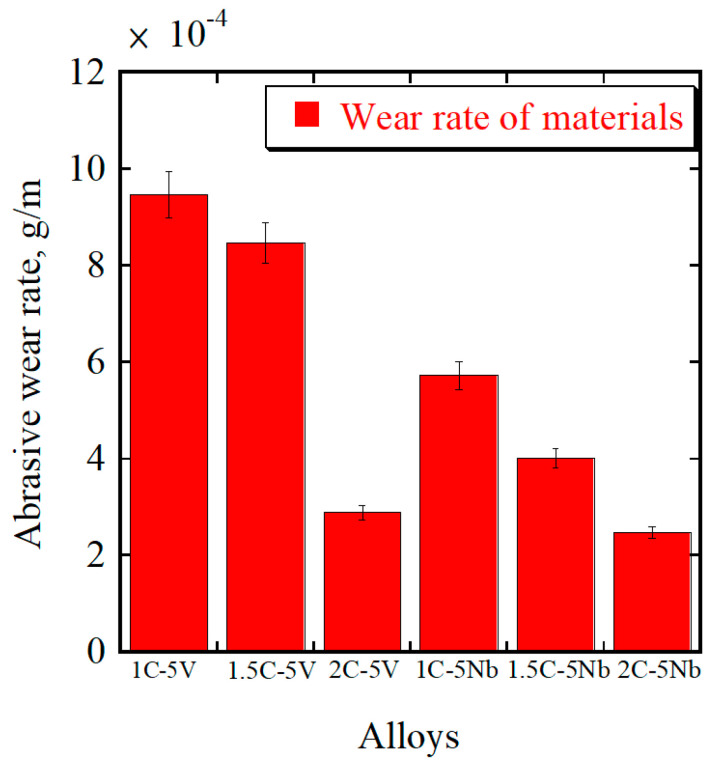
The abrasive wear rates for both alloys with different percentages of C content.

The average abrasive wear rate of each alloy is presented in a bar chart (Figure 8) with six repetitions of the test being performed. In the case of the 5V alloy, the wear rate decreased significantly as the percentage of C increased. The higher CVF and hardness values of the alloy as the amount of C increased probably contributed to the better wear resistance result. Therefore, 2C-5V has better wear resistance (wear rate: approximately 2.89 × 10*^−^*^4^ g/m), while the worst is 1C-5V (wear rate: approximately 9.47 × 10*^−^*^4^ g/m). The same condition was also implied in the case of the 5Nb alloy, where a higher percentage of C provided a better wear resistance result. Thus, the material with better wear resistance behavior is 2C-5Nb (wear rate: approximately 2.48 × 10*^−^*^4^ g/m) and the worst is the 1C-5Nb alloy (wear rate: approximately 5.73 × 10*^−^*^4^ g/m). This result is in agreement with several previous studies that determined that the greater the CVF and strength of the material, the better the wear resistance [7,27,29,35].

However, it is interesting to observe the comparison of both alloys (5V and 5Nb) with the same percentage of C. In the previous section, we clearly showed that there was no significant CVF difference between the 5V and 5Nb alloys. Moreover, the hardness of each alloy was also relatively similar. Meanwhile, the wear rate of 1C-5Nb was much lower than 1C-5V. In the case of 1.5C, the wear rate of 5Nb was also much lower compared to the 5V alloy. A minor difference can be observed for the case of 2C, where the wear rate of 5Nb was slightly lower than 5V. This means that materials with the same CVF and hardness values do not always guarantee the same wear resistance level. On the other hand, it can be argued that the CVF and hardness values are not always the main indicators for evaluating the wear performance of a material, which contrasts with the results of several previous studies [4,17,29].

The size of NbC was larger than that of VC due to the larger atomic radius of Nb compared to V. In addition, the small amount of Mo together with Nb occupying the area of NbC also effectively enlarged the carbide’s size. Therefore, the carbide size should also be an important factor in this study, rather than only the CVF or hardness values. We also investigated the wear mechanism to obtain a more comprehensive understanding of it. However, from these results, it can be observed that the 2C-5Nb alloy has a superior abrasive wear resistance behavior, while the worst is evident for the 1C-5V alloy.

### 3.3. Three-Body Abrasive Wear Mechanism

**Figure 9:**
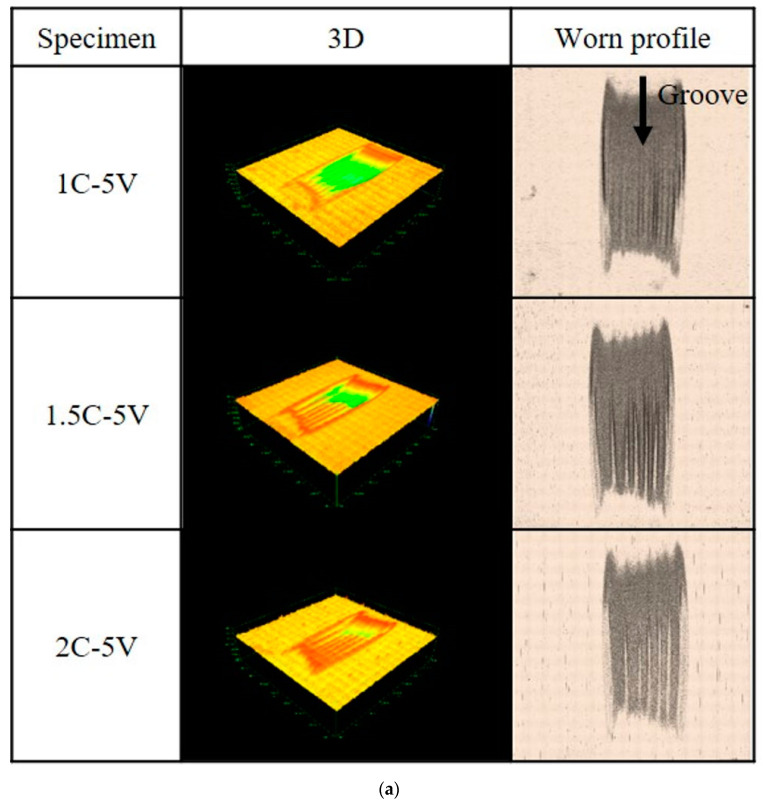

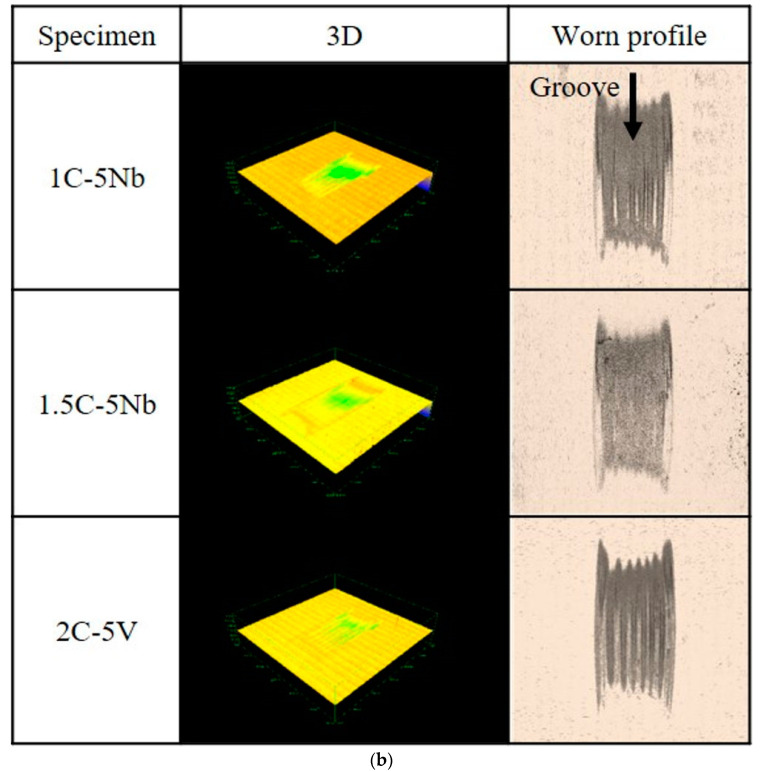
Investigation of the worn surface of each alloy using laser technology. (**a**) The 3D and worn profile of 5V-5Cr-5Mo-5W-5Co-Fe. (**b**) The 3D and worn profile of 5Nb-5Cr-5Mo-5W-5Co-Fe.

**Figure 10:**
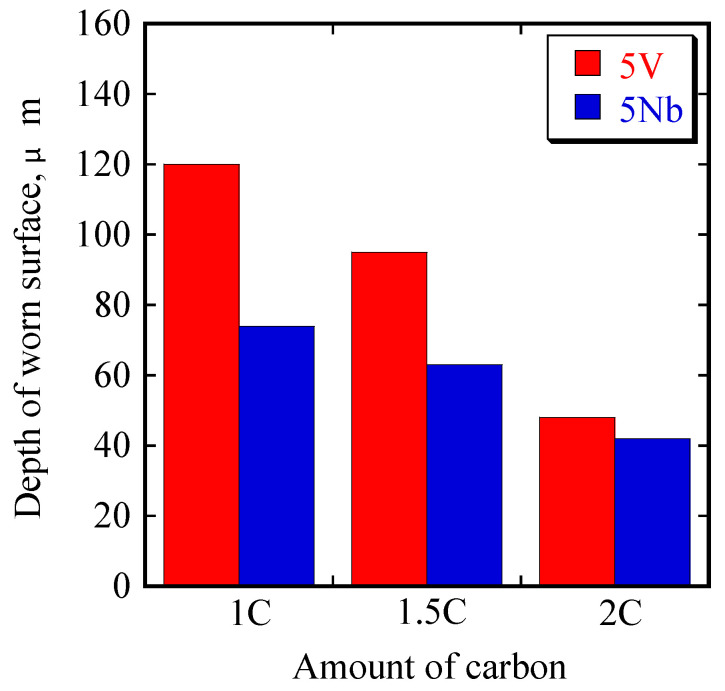
Depth values of worn surfaces of the two alloys with different C additions.

The worn surfaces and cross-sections of the most abraded surface of each alloy were observed to determine the three-body abrasive wear mechanism results. The surface of each alloy following testing using Optelics hybrid is presented in Figure 9. It can be observed that there is a groove on the worn surface presented as three abrasive wear tracks. We obtained the depth of the worn surface, and the results are presented in a bar chart in Figure 10. We can observe that the worn surface becomes shallower as the amount of C increases in the case of the 5V alloy. The deepest worn surface belongs to 1C (approximately 120 µm), while the shallowest surface belongs to 2C (approximately 48 µm). The same trend was also observed in the case of 5Nb. The deepest wear surface belongs to 1C-5Nb (approximately 77 µm) and the shallowest belongs to 2C (approximately 41 µm). In addition, it was observed that the wear surface depth of the 5Nb alloy was shallower than that of the 5V alloy with the same amount of C. Moreover, there was only a slight difference in the wear surface depth values between the 2C-5V and 2C-5Nb alloys. This condition relates to the wear performance of the alloy in that the alloy with the best wear resistance (2C-5Nb) has the shallowest wear surface, while the worst wear resistance (1C-5V) is connected to the deepest wear surface.

**Figure 11:**
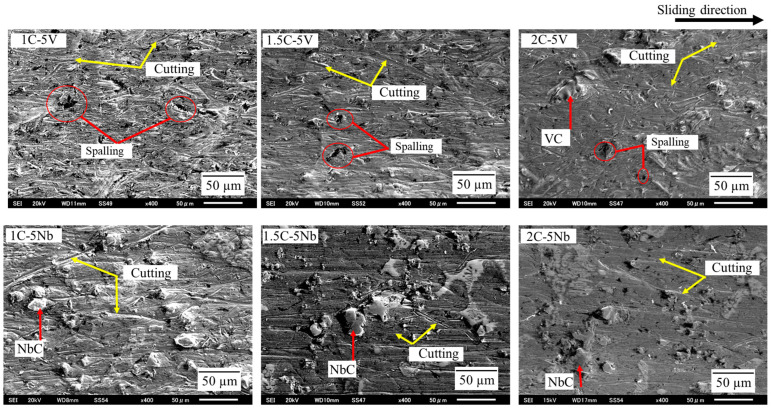
Investigation of abrasive wear mechanisms through worn surfaces using SEM.

A more detailed description of the worn surface (Figure 11) can be investigated using SEM. In the case of the 5V alloy, it could be observed that micro-cutting, mostly localized to the matrix region, occurred. This was due to the reduced hardness of the matrix than silica sand as an abrasive particle (1100 HV). However, the micro-cutting progressively disappeared as the percentage of C addition increased, which was a result of the increase in the microhardness (matrix). Due to the increase in the CVF as the amount of C increases, as previously described, the carbide spacing became denser, making it difficult for the silica sand to cut the matrix area. This might have also contributed to the decrease in the micro-cutting behavior as the percentage of C increased. In addition, spalling was also observed which was mostly localized to the VC carbide region. However, it was difficult to observe this process as the amount of C increased. Additionally, some VC carbides still remained firmly standing on the worn surface of 2C. In the case of 5Nb, the presence of micro-cutting was observed on the alloy matrix, which became more difficult to observe as the percentage of C increased. Meanwhile, the presence of spalling was difficult to observe on the surface of the 5V alloy. Additionally, NbC carbides appeared to stand firmly on the worn surface of each alloy.

**Figure 12:**
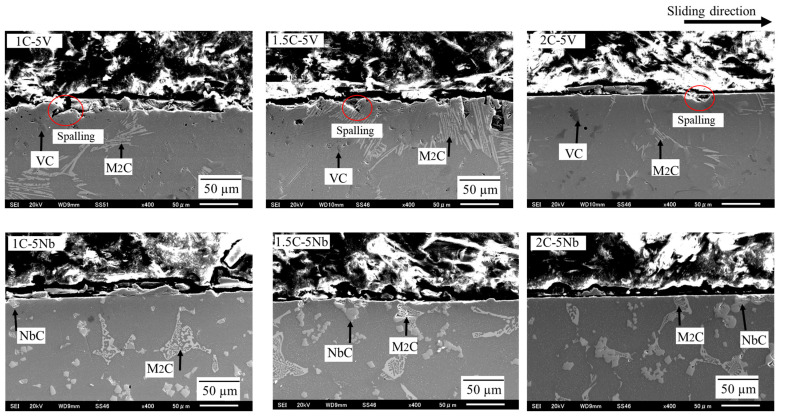
Investigation of abrasive wear mechanisms via the cross-section of the worn surface of each alloy studied.

From a cross-sectional viewpoint (Figure 12), a fairly similar condition can be observed on the wear surfaces of each alloy. The VC carbides appear to immediately peel off during the test, which may be due to their small size, as in the case of the 5V alloy. However, this tendency is suppressed as the percentage of C addition increases. When comparing the 5V alloy to 5Nb, a better cross-section can be obtained and no areas of spalling can be observed in the cross-section of the 5Nb alloy. This may be due to the larger size of NbC carbides than VC. In some previous studies [11,13,20], it has been reported that larger carbide sizes have lower wear resistance properties due to their brittleness. However, this condition is unlike the one observed in this study. These different results may be due to the different chemical compositions of the carbides. In addition, a better cross-section can be observed when there is an increase in the percentage of C addition in the 5Nb alloy.

When the silica sand abraded the surface of the alloy, it would cut the matrix area and the carbide area leaving evidence of micro-cutting marks and spalling. This means that the increased hardness of the alloy would provide the matrix with better protection against the cutting phenomenon and then effectively reduce the tendency of spalling on the carbides. This condition meant that the 2C alloy had greater wear resistance than 1C or 1.5C in the case of 5V. However, spalling was also effectively suppressed by increasing the carbide’s size, which meant that the 2C-5Nb alloy presented the best abrasive wear resistance behavior out of all the alloys. Therefore, the carbide’s size is very important to consider, instead of the CVF and the level of hardness when evaluating the alloy’s abrasive wear behavior. 

## 4. Conclusions

The wear behavior characteristics of 5V/5Nb-5Cr-5Mo-5W-5Co-Fe multicomponent cast alloys with varying C percentages under three-body abrasive wear conditions were examined in the present study. The results obtained can be summarized as follows:

1. There are several types of carbides precipitated in the microstructures of the studied alloys, for which the shape and type strongly depend on the chemical composition.
2. The CVF increases as the amount of C addition increases. Therefore, the carbide’s space might be denser, making it difficult for the silica sand to abrade the matrix area leading to improved wear resistance properties.
3. There is no significant difference in the hardness between the two alloys (5V and 5Nb) with the same amount of C due to relatively similar CVF values.
4. The NbC carbide’s larger size, in comparison to VC, produced better wear resistance. Therefore, the 2C-5Nb alloy presents the best three-body abrasive wear resistance behavior among all the alloys studied.

## Figures and Tables

**Table 1:** The chemical compositions of the studied alloys (wt.%).

Alloy	C	Cr	Mo	W	Co	V	Nb	Fe
1C-5V	1.12	4.89	5.02	4.97	4.89	5.15	-	Bal.
1.5C-5V	1.52	4.85	5.10	5.03	4.96	5.01	-	Bal.
2C-5V	1.92	4.84	5.07	4.90	4.42	4.79	-	Bal.
1C-5Nb	0.97	5.06	5.21	5.10	5.07	-	5.06	Bal.
1.5C-5Nb	1.39	5.22	4.97	5.14	5.07	-	4.89	Bal.
2C-5Nb	1.90	5.03	5.01	5.03	4.87	-	4.91	Bal.

## Data Availability

The data presented in this study are available upon request from the corresponding author.
